# Diagnostic Value of Salivary miRNA in Head and Neck Squamous Cell Cancer: Systematic Review and Meta-Analysis

**DOI:** 10.3390/ijms22137026

**Published:** 2021-06-29

**Authors:** Jeong-Wook Kang, Young-Gyu Eun, Young-Chan Lee

**Affiliations:** Department of Otolaryngology-Head and Neck Surgery, Kyung Hee University School of Medicine, Seoul 05278, Korea; simbody@naver.com (J.-W.K.); ygeun@hanmail.net (Y.-G.E.)

**Keywords:** miRNA, head and neck, cancer, biomarker, saliva, systemic review, meta-analysis

## Abstract

Several studies have highlighted the diagnostic potential of salivary microRNA (miRNA) in head and neck squamous cell cancer (HNSCC). The purpose of this meta-analysis was to summarize published studies and evaluate the diagnostic accuracy of salivary miRNA in HNSCC detection. In this meta-analysis, we systematically searched PubMed, EMBASE, and Cochrane Library databases for studies on miRNA and HNSCC diagnosis. Pooled sensitivity, specificity, and diagnostic odds ratio (DOR) with a summary receiver-operating characteristic curve were calculated using a bivariate random-effect meta-analysis model. Furthermore, subgroup analyses were conducted to explore the main sources of heterogeneity. Seventeen studies from ten articles, including 23 miRNA and a total of 759 subjects, were included in this meta-analysis. The pooled sensitivity and specificity of salivary miRNA in the diagnosis of HNSCC were 0.697 (95% CI: 0.644–0.744) and 0.868 (95% CI: 0.811–0.910), respectively. The overall area under the curve was 0.803 with a DOR of 12.915 (95% CI: 9.512–17.534). Salivary miRNAs are a promising non-invasive diagnostic biomarker with moderate accuracy for HNSCC. These results must be verified by large-scale prospective studies.

## 1. Introduction

Many biomarkers have been proposed that have a significant impact on the diagnosis and prognosis of head and neck squamous cell cancer (HNSCC) patients, but most of them are insufficiently validated for use in clinical practice [1]. Therefore, there is a need to discover new non-invasive biomarkers for the diagnosis and prognosis of HNSCC.

MicroRNAs (miRNAs) are small, highly-conserved non-coding RNAs with a length of approximately 18 to 25 nucleotides. miRNAs bind to the 3${}^{\prime }$-untranslated region (3${}^{\prime }$-UTR) of target messenger RNAs (mRNAs) and regulate gene expression post-transcriptionally through RNA degradation and/or translational inhibition [2]. miRNAs influence several biological processes that make up the hallmarks of cancer, including proliferation signals, cell death and metastasis [3]. Thus, aberrant miRNA expression is a characteristic feature of human cancer, and the identification of aberrant miRNAs and their respective targets can provide potential diagnostic and prognostic tumor biomarkers as well as novel therapeutic strategies for cancer treatment. Moreover, miRNA expression patterns are cell- and tissue-specific, so miRNAs could be used as biomarkers to diagnose various types of cancer and could also predict the survival and prognosis of patients.

A recently published meta-analysis revealed that the most upregulated miRNAs in HNSCC are miR-21 and miR-93, whereas miR-9, miR-203, miR-218 and miR-375 are downregulated [4]. The meta-analysis of the diagnostic value of miRNA in head and neck cancer (HNC) demonstrated that noninvasive miRNAs are a promising diagnostic tool with moderate accuracy for HNC diagnosis [5]. MiRNAs are also present in various body fluids, including saliva, and their expression levels are stable and consistent over time within an individual [6,7].

As a sample of a liquid biopsy, saliva offers many advantages over blood in that it is easy to collect, safe, non-invasive, and cost-effective [8,9]. Therefore, the reconstruction of the microRNA profile in the saliva, which is in contact with the tumorous tissue, could be exploited as a valuable source of biomarkers for HNSCC diagnosis and clinical prediction. For this reason, several studies of HNSCC, especially those including oral cancer, have investigated miRNA expression in the saliva. Previous narrative and systematic reviews of meta-analysis studies have highlighted the diagnostic value of circulating miRNAs for HNSCC as well as their potential role as prognostic factors in patients with oral cancer [10,11,12,13]. The diagnostic value can be measured quantitatively by the diagnostic odds ratio (DOR). The DOR ranges from zero to infinity. DOR value one means that a diagnostic test provides no information. The higher the DOR value, the better the diagnostic efficacy [14]; however, meta-analysis data of the diagnostic value of salivary miRNAs in HNSCC are still insufficient. Therefore, we conducted a systematic and comprehensive meta-analysis of all eligible studies to explore the overall diagnostic values of salivary miRNAs as promising biomarkers for HNSCC cancer detection.

## 2. Results

### 2.1. Literature Search

**Figure 1:**
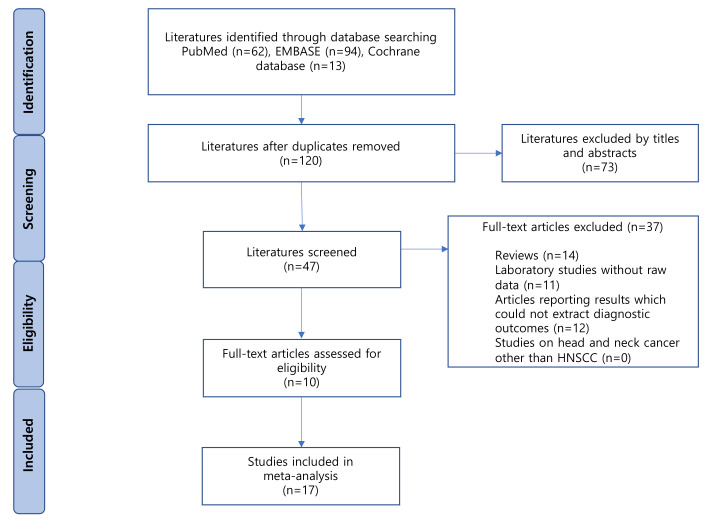
Flowchart. HNSCC; head and neck squamous cell cancer.

A flow diagram of the selected studies is shown in Figure 1. A total of 193 articles were identified from the databases, of which 73 duplicates were excluded. Of the remaining 120 articles, 73 were excluded based on their titles and abstracts. Of the 47 remaining articles, 37 were excluded for the following reasons: reviews (*n* = 14); laboratory studies without raw data (*n* = 11); articles reporting results without the possibility of extracting diagnostic outcomes (*n* = 12). Finally, ten eligible studies were included in our meta-analysis [15,16,17,18,19,20,21,22,23,24].

### 2.2. Study Characteristics and Quality Assessment

**Figure 2:**
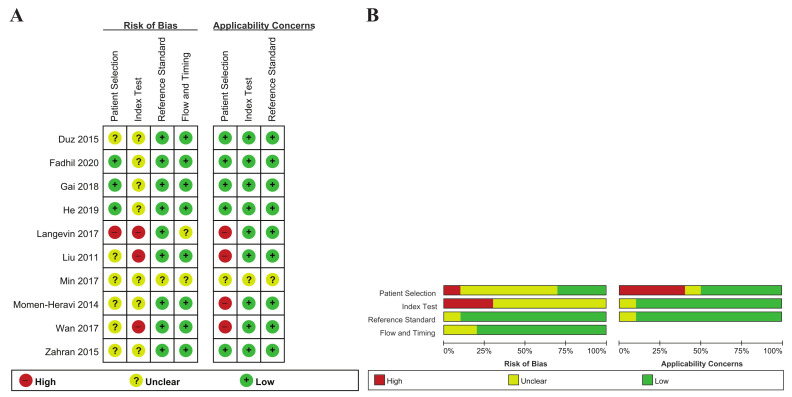
(**A**) Risk of bias and applicability concerns graph. Review author judgements about each domain are presented as percentages across the included studies; (**B**) Risk of bias and applicability concerns summary. Review author judgements about each domain for each study included.

The ten articles included here were comprised of 17 individual studies, including 23 unique miRNAs, and 759 subjects (443 HNSCC patients and 316 healthy controls) (Table 1). All of the studies were published between 2011 and 2020. Of the 17 studies, 15 analyzed single miRNAs, and the remaining two used multiple miRNA assays. Eight countries were included in the ten included articles: two studies were conducted in both Australia and the United States, and one in each of China, Italy, Korea, Turkey, Saudi Arabia and Taiwan. Of the 17 studies, 11 were limited to patients with oral squamous cell carcinoma (OSCC), and the remaining six studies included all patients with HNSCC. The target miRNA was increased in 12 studies and decreased in five. In most of the articles, the miRNA was extracted from whole saliva or supernatant, while He et al. (2019), Gai et al. (2018), and Langevin et al. (2017) analyzed miRNAs extracted from salivary exosomes. Salivary miRNA detection was conducted with RT-qPCR, except for in Langevin et al. (droplet digital PCR). The quality assessment results of the included studies using RevMan 5.3 are based on the QUADAS-2 evaluation tool, as shown in Figure 2.

### 2.3. Meta-Analysis

**Figure 3:**
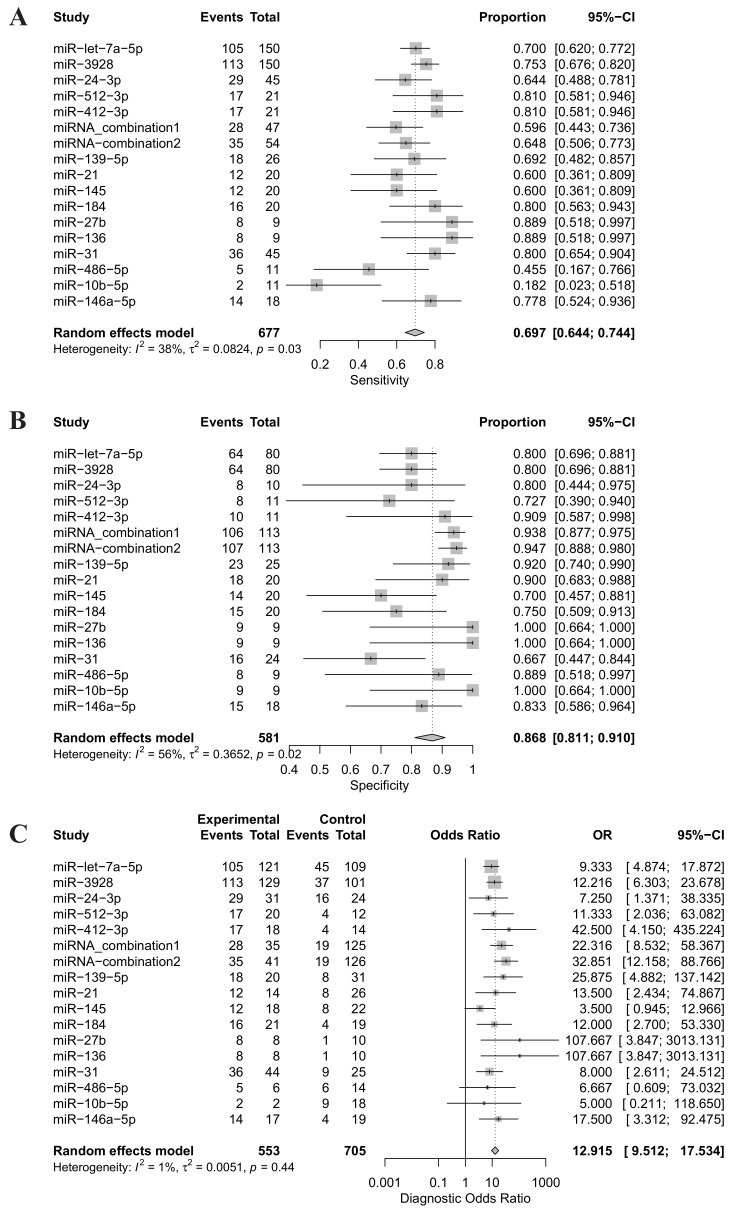
(**A**) Forest plot of the sensitivity of salivary miRNAs for distinguishing head and neck squamous cell cancer patients from healthy controls; (**B**) Forest plot of the specificity of salivary miRNAs for distinguishing head and neck squamous cell cancer patients from healthy controls; (**C**) Forest plot of diagnostic odds ratio of salivary miRNAs for distinguishing head and neck squamous cell cancer patients from healthy controls. miRNA-combination1 includes miRNA-9, -127, -134, -191, -222 and -455; miRNA-combiation2 includes miRNA-9, -134, -210, -455, and -196b; CI, Confidential interval; OR, odds ratio.

**Figure 4:**
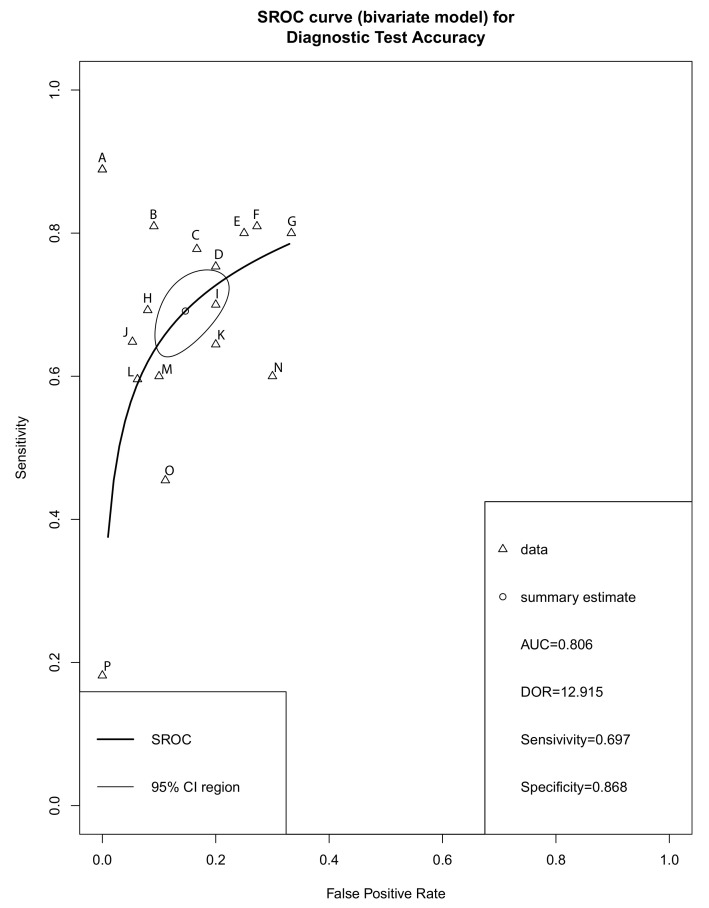
Summary receiver operator characteristic (SROC). A, miR-27b; B, miR-412-3p; C, miR-146a-5p; D, miR-3928; E, miR-184; F, miR-512-3p; G, miR-31; H, miR-139-5p; I, miR-let-7a-5p; J, miRNA-9, -134, -210, and -455 and -196b; K, miR-24-3p; L, miRNA-9, -127, -134, -191, -222 and -455; M, miR-21; N, miR-145; O, miR-486-5p; P, miR-10b-5p; CI, Confidential interval; AUC, area under the ROC curve; DOR, Diagnostic odds ratio.

Forest plots of sensitivity, specificity and DOR for salivary miRNA detection in HNSCC diagnosis were conducted (Figure 3). Heterogeneity analysis revealed I2 values of 38% (*p* = 0.02) for sensitivity and 56% (*p* = 0.02) for specificity, indicating significant heterogeneity. On the other hand, the DOR for salivary miRNA detection showed no significant heterogeneity, with an I2 value of 1% (*p* = 0.44). Therefore, the random-effect model was selected for this study. The pooled sensitivity and specificity were 0.697 (95% CI: 0.644–0.744) and 0.868 (95% CI: 0.811; 0.910), respectively. The pooled DOR was 12.915 (95% CI: 9.512–17.5340. The summary receiver operating characteristic (SROC) curve was plotted with an AUC value of 0.803, indicating a moderate diagnostic accuracy for salivary miRNA assays in HNSCC detection (Figure 4).

### 2.4. Subgroup Analysis

Subgroup analyses were conducted to evaluate the source of heterogeneity between studies, which is based on the source of miRNA extraction (whole saliva/salivary exosome), expression (up-regulation or down-regulation) and miRNA profiling (single miRNA/multiple miRNAs) (Table 2). With regard to the source of miRNA extraction, the miRNAs detected from the whole saliva group had a relatively higher sensitivity, specificity and DOR compared with the exosome group, with a sensitivity of 0.713 (95% CI: 0.673–0.749) versus 0.612 (95% CI: 0.387–0.797), specificity of 0.867 (95% CI: 0.800–0.915) versus 0.860 (95% CI: 0.734–0.932), and DOR of 13.680 (95% CI: 9.277–20.172) versus 10.477 (95% CI: 4.138–26.525). In the subgroup analysis based on expression, specificity (0.884 [95% CI: 0.814–0.930]) and DOR (16.187 [95% CI: 10.480–25.001]) in the upregulation group were higher than in the down-regulation group. Finally, in the analysis based on miRNA profiling, the sensitivity and specificity was similar between the single and multiple miRNA groups, but the multiple miRNA group had a much higher DOR than the single miRNA group, with a DOR of 26.902 (95% CI: 13.479–53.6930) versus 10.837 (95% CI: 7.748–15.157).

### 2.5. Publication Bias

**Figure 5:**
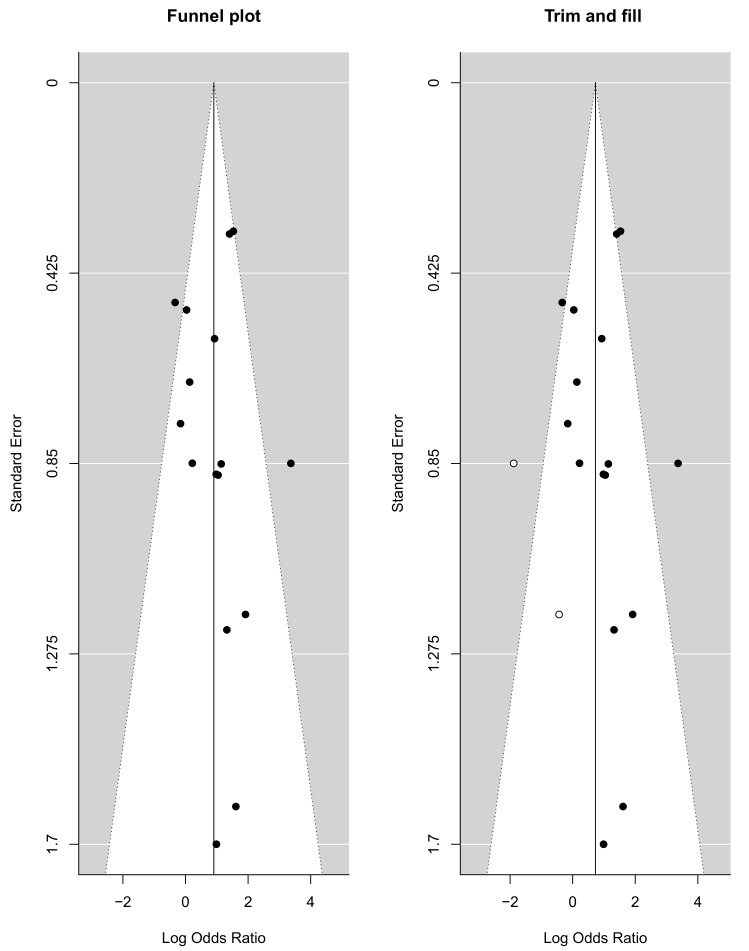
Funnel plot with trim and fill method.

The funnel plots provided in Figure 5 visually represent the likelihood of publication bias in this systematic review and meta-analysis study. The slope coefficient was associated with a *p* value of 0.386, which suggested no publication bias. The funnel plot for the DOR was observed to be slightly asymmetric, with a more significant number of studies falling on the right of the line of mean effect. Trim and fill were used to impute possible missing studies, which led to the imputation of two missing studies, adjusting the point estimate and its 95% CI from 0.905 (95% CI: 0.431–1.380) to 0.727 (95% CI: 0.210–1.243) after imputation.

## 3. Discussion

When oncogenic miRNA is overexpressed, tumor development is induced through the inhibition of tumor suppressor genes and/or genes that regulate cell differentiation or apoptosis. miR-372/373, miR-17-92, miR-21 and miR-155 are known to be involved in this process [3,25]. Conversely, miRNAs can act as tumor suppressors against tumor development (e.g., let-7 and miR-15a/16-1, miR-34a, miR-143/145) [26,27]. In particular, some miRNAs have oncogenic or tumor suppressor roles depending on the type of tissue they are expressed in [26]. As a result, specific miRNA expression patterns can not only differentiate cancer cells from normal cells, but can also identify the tissue in which the primary tumor originated and can be an early diagnostic marker of HNSCC [27,28,29]. Previous studies have shown that human molecules exhibit increased stability in saliva [7]. In particular, using cell-free saliva, studies have confirmed that miRNAs are stable and abundantly found in the saliva and within exosomes [6]. Therefore, salivary miRNA expression could be used to discover signatures related to carcinogenesis and progression [30].

Our meta-analysis included ten eligible articles (17 studies) with 443 cancer patients and 316 healthy controls. The overall analysis showed a moderate diagnostic accuracy of salivary miRNAs with an AUC of 0.803, sensitivity of 0.697 and specificity of 0.868. The pooled DOR in our meta-analysis was 12.915, which indicates a moderate level of accuracy to discriminate between HNSCC patients and healthy controls. To the best of our knowledge, this is the first meta-analysis to report the diagnostic role of salivary miRNA as a promising biomarker in HNSCC.

According to a meta-analysis by Tian et al., which included 23 studies, the pooled sensitivity, specificity and AUC were 0.759, 0.773 and 0.832, respectively, indicating a relatively high diagnostic accuracy of miRNAs in differentiating OSCC patients from healthy controls [31]. However, they did not evaluate the diagnostic accuracy of miRNAs in saliva because this analysis included results of miRNAs in the plasma and tissue as well as the saliva. Another meta-analysis reported that the pooled sensitivity and specificity of blood and salivary miRNAs in the diagnosis of OSCC were 0.78 and 0.82, respectively. The overall AUC was 0.91, with a DOR of 21.46. This study also only included the results of miRNAs in the saliva and blood of patients with OSCC [13]. Compared with these previous meta-analysis results, in our study, the pooled sensitivity and AUC of saliva miRNA in HNSCC were slightly lower, but the specificity was higher, confirming that miRNA is a relatively promising diagnostic biomarker. The diagnostic results were superior to the pooled sensitivity and specificity of DUSP-1 and S100P in the saliva of OSCC patients [32]. Salivary miRNAs have the ability to discriminate HNSCC from healthy controls with a DOR of 12.915, suggesting that miRNAs are promising molecular markers for the early identification of HNSCC.

We conducted subgroup analyses based on the source of miRNA extraction, miRNA expression and miRNA profiling. In the subgroup analysis based on the source of extraction, miRNA retrieved from whole saliva group or from the supernatant of saliva were compared. For cancer detection in previous meta-analysis studies, no differences have been reported between miRNAs extracted from the whole saliva and supernatant [33]. However, there has not been any comparisons of the diagnostic values of salivary exosomal miRNAs and salivary miRNAs. This meta-analysis confirmed that salivary miRNAs are superior in sensitivity, specificity and DOR. The lack of diagnostic value of exosomal miRNAs may be due to few published articles using this retrieval method. In addition, although previous studies have shown that exosome isolation enriches salivary miRNA biomarkers of tumor cells, exosome isolation remains challenging [34]. Because saliva is made up of a complex mixture of secretions from several tissues, foreign substances can interfere with the detection of biomarkers in exosomes [35]. On the other hand, subgroup analysis according to miRNA expression and miRNA profiling showed inconsistent results in sensitivity, specificity and DOR, making it difficult to prove a significant difference.

Since heterogeneity often exists in meta-analyses of diagnostic accuracy data, possible causes that could contribute to the inconsistency in accuracy estimates across the study were investigated and a random-effect model was used. Interestingly, the I2 value of the heterogeneity test of DOR in our study was 1%, indicating no significant heterogeneity. Furthermore, the funnel plot asymmetry test showed no evidence of publication bias (*p* = 0.386).

Our meta-analysis has some limitations. First, because all of the studies included in our analysis were case-control studies, the risk bias of quality assessment was unclear. Second, in integrating the results of the included articles, there may have been others, such as various patient stages, HNSCC subtype, HPV status, miRNA normalization methods and cutoffs, which were not accounted for. Third, our data suggest that several types of miRNAs have excellent accuracy and clinical value, but few overlapping miRNAs were found between studies, and the most efficient method for combining miRNAs has not yet been identified. Finally, despite the “mathematically” obtained values of sensitivity and specificity in this meta-analysis, the diagnostic accuracy of each miRNA as a promising biomarker could not be validated.

## 4. Materials and Methods

### 4.1. Search Strategy

We conducted a meta-analysis according to the guidelines of preferred reporting items for systematic review and meta-analysis (PRISMA) [36]. Up until 3 June 2020, a comprehensive literature search was conducted in the PubMed, EMBASE, and Cochrane Library databases. The search strategy was as follows: (MicroRNA OR miRNA OR miR) AND (cancer OR tumor OR neoplasm OR tumor OR malignant OR carcinoma OR Squamous Cell Carcinoma OR SCC) AND (head and neck OR larynx OR oropharynx OR hypopharynx OR nasopharynx OR oral and cavity OR mouth OR laryngeal OR pharyngeal OR sinus OR sinonasal OR tongue) AND (Saliva OR Salivary) AND (diagnosis OR sensitivity OR specificity OR ROC curve) AND (marker OR biomarker).

### 4.2. Study Included/Excluded Criteria

Two authors (Lee and Kang) independently conducted the literature search and study selection method. Studies were included if they met the following inclusion criteria: (1) miRNAs were used for diagnosis. (2) Patients with HNSCC were confirmed by pathological biopsy as a reference standard. (3) Saliva samples from patients with HNSCC were used and healthy controls were included, whether patients with benign disease or healthy people. (4) Sufficient information was reported to construct a 2 × 2 table for calculating true positives (TP), true negatives (TN), false positives (FP), and false negatives (FN). The exclusion criteria were as follows: (1) Articles were reviews, comments, or case reports. (2) Articles lacked sufficient data for extraction. (3) Articles presented experiments on animal or cell lines.

### 4.3. Data Extraction and Quality Assessment

Two authors individually extracted data from selected studies. The full texts of the included studies were reviewed and the following data items were extracted: (1) Characteristics of studies: the first author’s family name, publication date, country of study, sample size, age and sex data, source of miRNA extraction, method of salivary miRNA detection, detected miRNAs, and miRNA expression. (2) Diagnostic outcomes: sensitivity, specificity, cutoff, area under the ROC curve (AUC), and information needed for quality assessments. If one study reported diagnostic results for two or more different types of miRNA, each miRNA test was considered as an independent study. In addition, the results of multiple miRNA combination assays were considered as independent study data. The quality of the selected studies was assessed independently by two reviewers using the revised quality assessment of diagnostic accuracy studies (QUADAS-2) checklist. This checklist comprises four domains: patient selection, index test, reference standard, and flow and timing for a transparent rating of bias and the applicability of primary diagnostic accuracy studies [37].

### 4.4. Statistical Analysis

We performed quantitative synthesis of the data using R software (R package version 4.0.0). We conducted statistical univariate and bivariate analyses to summarize the sensitivity, specificity, and DOR, together with their 95% confidence intervals (95% CIs). The package commands in the R software were “metaprop” and “metabin” for sensitivity, specificity, and DOR; forest for forest plot; and reitsma of “mada” for a summarized receiver-operating characteristic (ROC) curve. The pooled sensitivity and specificity were calculated to evaluate the summary receiver operator characteristic curve (SROC) and the area under the curve (AUC). Most analyses used random-effects models rather than fixed-effects models because of the dissimilarity of the enrolled studies. An inspection of heterogeneity was conducted using Cochran’s Q test and Higgins I-squared statistic, where an I2 value greater than 50% indicated heterogeneity between the studies [38]. A subgroup analysis was used to explore the sources of heterogeneity. Funnel plots with trim and fill methods were used to estimate publication bias. The trim and fill method estimated the number of potential missing studies and recalculated the effect estimate. A two-tailed *p* value less than 0.05 was considered statistically significant.

## 5. Conclusions

This meta-analysis suggests that salivary miRNA could be a promising non-invasive biomarker for HNSCC diagnosis. Although several miRNAs show excellent diagnostic accuracy, further research, including large-scale prospective studies in multi-center HNSCC cohorts, is needed to validate its application in clinical practice.

## Figures and Tables

**Table 1:** Extracted data for performing the statistical analysis.

Author	miRNA Profiling	Country	Healthy Controls			HNSCC Patients			Cancer Spectrum	Sensitivity	Specificity	AUC (SE)	Change in Expression	Source of miRNA	Method of miRNA Detection
			N	Sex (M/F)	Mean Age (SD or Range)	N	Sex (M/F)	Mean Age (SD or Range)							
Fadhil et al. (2020)	miR-let-7a-5p	Australia	80	35/45	56.6	150	90/60	60.5	HNSCC	0.7	0.8	0.85	down	Saliva (supernatant)	RT-qPCR
Fadhil et al. (2020)	miR-3928	Australia	80	35/45	56.6	150	90/60	60.5	HNSCC	0.75	0.8	0.74	down	Saliva (supernatant)	RT-qPCR
He et al. (2019)	miR-24-3p	China	10			45			OSCC	0.644	0.8	0.738	up	Exosome	RT-qPCR
Gai et al. (2018)	miR-512-3p	Italy	11	05-6	61.64 (61.5–67.5)	21	09-12	65.75 (61–73)	OSCC	0.8	0.7	0.847	up	Exosome	RT-qPCR
Gai et al. (2018)	miR-412-3p	Italy	11	05-6	61.64 (61.5–67.5)	21	09-12	65.75 (61–73)	OSCC	0.8	0.95	0.871	up	Exosome	RT-qPCR
Wan et al. (2017)	miRNA-9, -127, -134, -191, -222, and -455	Australia	113	59/54	44.7 (11.4, 19–79)	47	34/13	64.3 (10.38, 42–91)	HNSCC	0.6	0.94	0.82	up	Saliva (supernatant)	RT-qPCR
Wan et al. (2017)	miRNA-9,-134, -210, -455, and -196b	Australia	113	59/54	44.7 (11.4, 19–79)	54	5-49	59.7 (11.4, 37–87)	HNSCC	0.65	0.95	0.8	up	Saliva (supernatant)	RT-qPCR
Langevin et al. (2017)	miR-486-5p	USA	9	05-4	36 (19–53)	11	02-9	58 (47–73)	HNSCC	0.45	0.89		up	Exosome	Droplet digital PCR
Langevin et al. (2017)	miR-10b-5p	USA	9	05-4	36 (19–53)	11	02-9	58 (47–73)	HNSCC	0.18	1		up	Exosome	Droplet digital PCR
Min et al. (2017)	miR-146a-5p	Korea	18			18			OSCC	0.78	0.85	0.9	up	Saliva	RT-qPCR
Duz et al. (2015)	miR-139-5p	Turkey	25	21-4	46.88 (3.63)	25	19-6	54.08 (3.63)	OSCC	0.7	0.9	0.805	down	Saliva (superantant)	RT-qPCR
Zahran et al. (2015)	miR-21	Saudi Arabia	20	11-9	51.1 (9.3, 37–65)	20	08-12	58 (9.2, 38–73)	OSCC	0.6	0.9	0.73	up	Saliva (supernatant)	RT-qPCR
Zahran et al. (2015)	miR-145	Saudi Arabia	20	11-9	51.1 (9.3, 37–65)	20	08-12	58 (9.2, 38–73)	OSCC	0.6	0.7	0.68	down	Saliva (supernatant)	RT-qPCR
Zahran et al. (2015)	miR-184	Saudi Arabia	20	11-9	51.1 (9.3, 37–65)	20	08-12	58 (9.2, 38–73)	OSCC	0.8	0.75	0.86	up	Saliva (supernatant)	RT-qPCR
Momen-Heravi et al. (2014)	miR-27b	USA	9	03-5	60.16 (9.57, 32–77)	9	01-8	60.66 (11.83, 41–78)	OSCC	0.8571	1	0.9643 (0.0443, 0.877–1.052)	up	Saliva (supernatant)	RT-qPCR
Momen-Heravi et al. (2014)	miR-136	USA	9	03-5	60.16 (9.57, 32–77)	9	01-8	60.66 (11.83, 41–78)	OSCC	0.8889	1	0.9683 (0.093, 0.8904–1.046)	down	Saliva (supernatant)	RT-qPCR
Liu et al. (2011)	miR-31	Taiwan	21	20-1	51.4 (8.4)	43	2-41	53.9 (9.4)	OSCC	0.8	0.65	0.82	up	Saliva (supernatant)	RT-qPCR

HNSCC; head and neck squamous cell cancer, OSCC; Oral squamous cell cancer, AUC; area under the ROC curve, SD; standard deviation, SE; Standard error.

**Table 2:** Subgroup analysis.

Subgroup	Sensitivity (95% CI)	Specificity (95% CI)	DOR (95% CI)
Source of miRNA extraction			
Whole saliva	0.713 [0.673; 0.749]	0.867 [0.800; 0.915]	13.680 [9.277; 20.172]
Exosome	0.612 [0.387; 0.797]	0.860 [0.734; 0.932]	10.477 [4.138; 26.525]
Expression			
Up	0.685 [0.595; 0.764]	0.884 [0.814; 0.930]	16.187 [10.480; 25.001]
down	0.721 [0.672; 0.765]	0.813 [0.755; 0.860]	10.564 [5.855; 19.059]
miRNA profiling			
Single miRNAs	0.710 [0.650; 0.764]	0.817 [0.773; 0.854]	10.837 [7.748; 15.157]
Multiple miRNAs	0.624 [0.526; 0.713]	0.942 [0.903; 0.966]	26.902 [13.479; 53.693]

CI; Confidential interval, DOR; diagnostic odds ratio.
